# Repetitive Traumatic Brain Injury and Development of Chronic Traumatic Encephalopathy: A Potential Role for Biomarkers in Diagnosis, Prognosis, and Treatment?

**DOI:** 10.3389/fneur.2012.00186

**Published:** 2013-01-17

**Authors:** Ryan C. Turner, Brandon P. Lucke-Wold, Matthew J. Robson, Bennet I. Omalu, Anthony L. Petraglia, Julian E. Bailes

**Affiliations:** ^1^Department of Neurosurgery, School of Medicine, West Virginia UniversityMorgantown, WV, USA; ^2^Center for Neuroscience, School of Medicine, West Virginia UniversityMorgantown, WV, USA; ^3^Department of Basic Pharmaceutical Sciences, School of Pharmacy, West Virginia UniversityMorgantown, WV, USA; ^4^Department of Pathology, University of CaliforniaDavis, CA, USA; ^5^Department of Neurosurgery, University of Rochester Medical CenterRochester, NY, USA; ^6^Department of Neurosurgery, NorthShore University Health SystemEvanston, IL, USA; ^7^Section of Neurosurgery, Department of Surgery, University of Chicago Medical CenterChicago, IL, USA

**Keywords:** chronic traumatic encephalopathy, CTE, TBI, biomarkers, imaging

## Abstract

The diagnosis of chronic traumatic encephalopathy (CTE) upon autopsy in a growing number of athletes and soldiers alike has resulted in increased awareness, by both the scientific/medical and lay communities, of the potential for lasting effects of repetitive traumatic brain injury. While the scientific community has come to better understand the clinical presentation and underlying pathophysiology of CTE, the diagnosis of CTE remains autopsy-based, which prevents adequate monitoring and tracking of the disease. The lack of established biomarkers or imaging modalities for diagnostic and prognostic purposes also prevents the development and implementation of therapeutic protocols. In this work the clinical history and pathologic findings associated with CTE are reviewed, as well as imaging modalities that have demonstrated some promise for future use in the diagnosis and/or tracking of CTE or repetitive brain injury. Biomarkers under investigation are also discussed with particular attention to the timing of release and potential utility in situations of repetitive traumatic brain injury. Further investigation into imaging modalities and biomarker elucidation for the diagnosis of CTE is clearly both needed and warranted.

## Introduction

Increasing awareness by both medical professionals and the lay community concerning the potential long-term effects of repetitive traumatic brain injury, such as chronic traumatic encephalopathy (CTE) and cognitive impairment (Guskiewicz et al., 2005; Gavett et al., 2010; Daneshvar et al., 2011), has led to the identification of a need for improved diagnostic and prognostic tests. Investigators have focused primarily on the use of various imaging modalities and development of blood- or CSF-based biomarkers. In the following sections we attempt to briefly review findings associated with CTE diagnosis, proposed disease pathophysiology, and how these findings may potentially relate to imaging and/or biomarker discovery.

### Epidemiology and clinical presentation

Exposure to repetitive mild traumatic brain injury (mTBI) is a common occurrence in athletes on the playing field and soldiers on the battlefield. In fact, playing American football at higher levels results in documented exposure of up to 1400 impacts per season for select positions such as linemen with some players involved in both offense and defense sustaining nearly 2000 impacts (Stern et al., 2011). Similarly, mTBI has been identified as the most common combat-related injury in soldiers returning from present-day conflicts in Iraq and Afghanistan and consequently, has been described as the “signature injury of war” (Shenton et al., 2012). The diagnosis of mTBI remains particularly challenging due to the usual lack of abnormal findings on conventional CT and MR imaging (Shenton et al., 2012). Lack of a diagnostic test for mTBI is problematic considering the potential for both enduring cerebral effects (cognitive, neurophysiological, and clinical) and for identification of those at risk for development of CTE later in life.

Chronic traumatic encephalopathy represents a progressive neurodegenerative disease currently diagnosed only upon autopsy and subsequent neuropathological examination (Saulle and Greenwald, 2012). Despite the lack of specific diagnostic criteria required for pre-mortem clinical diagnosis, patients afflicted with CTE diagnosed post-mortem are often described as suffering behavioral, cognitive, and emotional changes or impairments prior to death (Gavett et al., 2011b; Omalu et al., 2011b; Saulle and Greenwald, 2012). Notably, symptom development occurs following a prolonged latency in most cases, although exceptions do exist (Gavett et al., 2011a; Omalu et al., 2011b). The tendency for a latent period creates a clear distinction between initial symptoms associated with traumatic brain injury (TBI) and the persistent, long-term degeneration, much like other neurodegenerative diseases such as Alzheimer’s disease (AD).

Chronic traumatic encephalopathy has been diagnosed in a broad spectrum of individuals with a history of head trauma, although the number of and severity of impacts is often unclear, ranging from athletes playing American football, soccer, hockey, boxers, and wrestlers to soldiers who have received battlefield injuries (Omalu et al., 2005, 2006, 2010a,b,c, 2011b; McKee et al., 2009; Baugh et al., 2012; Goldstein et al., 2012; Lakhan and Kirchgessner, 2012). Due to the variety of individuals afflicted by CTE, emerging evidence indicates that CTE is likely more common than previously thought (Stern et al., 2011; Baugh et al., 2012). Based on the experience of one group of investigators, a conservative estimate of lifetime prevalence of CTE in American football players is at least 3.7% (Saulle and Greenwald, 2012). Estimates of CTE prevalence in retired professional boxers have been as high as 20% (Lakhan and Kirchgessner, 2012). Considering the number of individuals actively engaged in contact sports such as football or exposed to explosive devices on the battlefield, it is clear that CTE represents a clear public-health risk. Much of the early work involving CTE diagnosis focused on concussion history but recent studies have documented a potential role of repetitive subconcussive blows as well (Omalu et al., 2010b; Baugh et al., 2012).

### Neuropathologic findings of CTE

#### Gross

Gross neuropathologic examination of the brain in individuals afflicted with CTE may produce a range of findings. The brain may appear grossly normal or may show minimal lobar cortical atrophy for age without remote cortical contusions or lacerations (Omalu et al., 2005). There may be other non-specific gross pathologic changes like fenestrations of the septi pellucidi, communicating ventriculomegaly, subcortical ganglionic atrophy, cerebellar folial atrophy, and pallor of the substantia nigra (Gavett et al., 2011b). In general, however, the CTE brain of non-boxers is grossly normal without evidence of focal traumatic brain injury.

The frequent lack of gross neuroanatomical changes observed in CTE is in striking contrast to dementia pugilistica, believed to represent a more severe form of CTE observed in boxers (Millspaugh, 1937; Corsellis et al., 1973; Adams and Bruton, 1989; Casson et al., 2006). Dementia pugilistica, described originally as “punch drunk” by Martland (1928), was characterized neuropathologically by Corsellis et al. (1973) based on a tetrad of findings: (1) abnormalities of the septum pellucidum; (2) cerebellar and other scarring of the brain; (3) degeneration of the substantia nigra; and (4) the presence of neurofibrillary tangles (NFTs) in a regional manner. While some controversy exists concerning identification of CTE, the work of Corsellis and colleagues is notable in that at no point is encephalopathy defined by the presence of a fenestrated or cavum septum pellucidum and equally important, the complete tetrad was not observed in a third of the cases characterized as dementia pugilistica (Casson et al., 2006).

#### Microscopic

Microscopic investigation of CTE has focused primarily on several factors: the presence of tau, amyloid, and presence of TAR DNA-binding protein 43 proteinopathy as well as low grade diffuse white matter rarefaction, microglial activation, and presence of reactive astrocytes (Gavett et al., 2011b). The presence of a tauopathy, whether it be in the form of neurofibrillary tangles (NFTs), neuropil threads (NTs), or glial tangles (GTs), is a defining feature of CTE. While other neurodegenerative diseases such as AD are also frequently defined and/or described by the presence of tau, CTE is clearly unique based on the topographic distribution of tauopathy (Gavett et al., 2011b). AD is characterized by a relatively uniform distribution of tau NFTs in layers containing large projection neurons, such as layers III and V (Gavett et al., 2011b). In contrast, CTE is exemplified by an irregular distribution of tau in more superficial cortical layers such as II and III. Similarly, the progressive topographic involvement of regions of the brain as seen in CTE differs from what is seen in other neurodegenerative diseases like AD. While neuritic amyloid plaques are seen in AD, neuritic amyloid plaques are not defining features of CTE, and are less frequently seen in CTE (Gavett et al., 2011b).

Another delineating factor between AD and CTE is the presence of neuritic beta amyloid (Aβ) plaques. Found extensively throughout the brains of those afflicted with AD, neuritic amyloid plaques are found in a minority of CTE sufferers. Diffuse and neuritic amyloid plaques are found in less than 40–45% of individuals with CTE (Blaylock and Maroon, 2011; Gavett et al., 2011b). Additionally, when found in CTE, amyloid plaques are more likely to be diffuse plaques and not the typical neuritic plaques that are diagnostic of AD (Gavett et al., 2011b). The role Aβ plays in CTE pathophysiology, and why it is present in some brains but not others, remains to be elucidated. Interestingly, amyloid precursor protein (APP), which can undergo cleavage to form Aβ, accumulates following axonal injury, and likely plays a role in plaque formation (Gavett et al., 2011b).

Chronic traumatic encephalopathy has been recently associated, in greater than 80% of cases, with accumulation of yet another phosphorylated protein aggregate, TDP-43 (Gavett et al., 2011b). In some cases, TDP-43 has been found extending into the anterior horns of the spinal cord, particularly in patients exhibiting motor neuron disease symptoms similar to those of amyotrophic lateral sclerosis (Gavett et al., 2011b; Stern et al., 2011). While TDP-43 proteinopathy occurs as a primary or secondary proteinopathy in a variety of neurodegenerative diseases, the significance of TDP-43 proteinopathy in CTE is presently not clear. The presence of another phosphorylated protein in aggregate form may indicate a shared process resulting in neurodegeneration following repetitive brain trauma (Gavett et al., 2011b). Being that TDP-43 has been suggested to mediate the response of the neuronal cytoskeleton following injury, brain trauma, and subsequent axonal injury may trigger a TDP-43-mediated process involved in neurodegeneration (Costanza et al., 2011).

The diversity of pathological findings associated with CTE, as represented by the varied findings presented above, has begun to be described with four distinct phenotypes emerging (Table 1; Omalu et al., 2011a). The significance of these phenotypes with regards to clinical correlate remains to be elucidated but it is becoming increasingly clear that CTE represents a diverse spectrum of disease, a finding consistent with the heterogeneous nature of injury history, genetic predisposition, and a variety of other factors.

**Table 1:** **The diversity of pathological findings in CTE has lead to the emergence of four distinct phenotypes**.

Phenotype	Cerebral cortex	Subcortical nuclei/basal ganglia	Brainstem	Cerebellum
#1	+Sparse to frequent NFTs and NTs	+With or without NFTs and NTs	+Sparse to frequent NFTs and NTs	+No NFTs and NTs
	+No diffuse amyloid plaques	
#2	+Sparse to frequent NFTs and NTs	+With or without NFTs and NTs	+Sparse to frequent NFTs and NTs	+No NFTs and NTs
	+Sparse to frequent diffuse amyloid plaques	
#3	+None to sparse NFTs and NTs	+None to sparse NFTs and NTs	+Moderate to frequent NFTs and NTs	+No NFTs and NTs
	+No diffuse amyloid plaques	
#4	+None to sparse NFTs and NTs	+None to sparse NFTs and NTs	+None to sparse NFTs and NTs	+No NFTs and NTs
	+No diffuse amyloid plaques	

*How each phenotype correlates to the clinical condition remains unclear but the varied disease findings are consistent with the diversity of clinical exposure and disease-related factors*.

### CTE pathogenesis and pathophysiology

The pathogenesis and pathophysiology of CTE remain unclear, as with many neurodegenerative diseases, but is believed to be a multifactorial process initiated by brain trauma. The development of CTE begins with subconcussive or concussive injury. The damage is progressive and often accelerated by the number of brain injuries that occur in an individual. Initially, mTBI causes diffuse axonal injury (DAI), which results in disruption of axonal transport and subsequent axonal swelling. The swelling causes a disconnection of the axons and later Wallerian degeneration (Johnson et al., 2012). This degenerative process, a portion of which is referred to in the literature as immunoexcitotoxicity, may lead to the development of CTE (Blaylock and Maroon, 2011). It is interesting to note that the abnormal tau and amyloid accumulations, which are seen in CTE are peptide derivatives of both membrane and cytoskeletal proteins, which are involved in traumatic axonal injury following concussive and subconcussive injury. This process is still relatively poorly understood clinically with post-mortem studies on young adults revealing that repetitive head injury is associated with the formation of NFTs and tau-based pathology surrounding vascular elements within the cortex (Geddes et al., 1999). Consequently, it is perhaps likely that microvascular damage plays a role in formation of the classical neuropathology associated with CTE. This is consistent with findings from classical literature exploring dementia pugilistica in which a large percentage of ex-boxers experienced perivascular hemorrhages with evidence of meningeal and/or subpial siderosis (Adams and Bruton, 1989). In addition to repetitive brain injury, there may be other identified factors that may contribute to or alter disease development, such as presence of certain genotypes (Omalu et al., 2010b). Notably, anabolic steroid use had been previously suggested as a potential contributing factor to CTE development but the use of exogenous anabolic steroids has been shown experimentally to not worsen mTBI (Mills et al., 2012).

While the precise pathway or mechanism via which repetitive brain trauma predisposes to CTE development is poorly elucidated, Blaylock and Maroon (2011) posit a logical process via which immunoexcitotoxicity mediates the transition. As part of this process, microglia are primed by initial impacts and with sustained trauma as well as aging, undergo phenotypic conversion from a non-destructive to destructive mode (Blaylock and Maroon, 2011; Saulle and Greenwald, 2012). Once phenotype switching occurs, this pro-inflammatory state can be maintained for prolonged periods, consistent with neurodegenerative processes and emergence of hyperphosphorylated tau. Similarly, mild injury has been demonstrated to damage axons due to degenerative processes, resulting in a progressive deterioration, rather than rapidly occurring axonal shearing. This furthers the notion of CTE as a chronic neurodegenerative process, clearly distinct from the immediate sequelae often associated with TBI (Blaylock and Maroon, 2011).

## Imaging Modalities for Repetitive Brain Injury and Chronic Traumatic Encephalopathy

A major concern for clinicians and researchers is how to detect small but important brain changes prior to the development of CTE symptoms so that preventative measures can be taken or treatments implemented, once available (Baugh et al., 2012; Saulle and Greenwald, 2012). The obvious short term deficits seen in concussive injuries such as loss of consciousness, post-traumatic amnesia, and altered mental status have been historically hard to quantify using traditional imaging modalities such as computed tomography (CT) and magnetic resonance imaging (MRI; Difiori and Giza, 2010; McCrory, 2011; Prichep et al., 2012). The difficulties and shortcomings of more commonly utilized imaging techniques have led researchers and clinicians to define concussion as a biomechanically induced brain injury with no gross anatomic lesions (Signoretti et al., 2011). Despite the lack of gross lesions, it is still necessary to look at diffuse and microscopic alterations in the brain. Until recently, imaging techniques were not sophisticated enough to detect the subtle changes that are the hallmark of DAI. Clinicians have previously been forced to rely on post-mortem tissue analysis to discover mechanisms of CTE pathology (Lakhan and Kirchgessner, 2012). With the invention and application of new imaging modalities, it is now possible to identify and investigate more of the pathological changes that are a consequence of mTBI. The following sections will highlight several of the new imaging modalities that are being used successfully to study various phenomena associated with mTBI and also promising modalities for further investigation of CTE. These modalities and potential strengths, as well as challenges, associated with each are summarized in Table 2.

**Table 2:** **Numerous imaging modalities may provide insight into the development or tracking of CTE**.

Imaging modality	Potential strengths	Potential weaknesses	References of interest
Diffusion tensor imaging (DTI)	+Radiologic and clinical deficits may correlate well	+May be time-intensive if tractography required	Henry et al. (2011a), Gavett et al. (2011a), and Rutgers et al. (2008)
	+Correlation between concussion history and DTI measures	
Functional magnetic resonance imaging (fMRI)	+Real-time assessment of brain activity and function	+Likely requires baseline scan for comparison	Gavett et al. (2011a), Talavage et al. (2010), McDonald et al. (2012), Breedlove et al. (2012), and Henninger et al. (2007)
	+Shown deficits in subconcussive injury	
Magnetic resonance spectroscopy (MRS)	+Insight into pathophysiology of CTE	+Difficulty distinguishing between natural changes with aging and those of injury	Henry et al. (2010), Kleindienst et al. (2005), Lin et al. (2010, 2012); Gavett et al. (2011a), and Tremblay et al. (2012)
	+Shown persistent changes in professional athletes	
	+Metabolites may correlate with pathology and function	
Positron emission tomography (PET)	+Identification of brain regions exhibiting decreased metabolism	+Exposure to radioactive isotopes may limit repeat scans	Venneti et al. (2007), Provenzano et al. (2010), Wagner (2012), and Small et al. (2006)
	+May measure tau deposition in CTE	
Single photon emission computer tomography (SPECT)	+Abnormalities in perfusion visualized in boxers with repeat trauma exposure	+SPECT fails to predict neuropsychological deficits	Kemp et al. (1995) and Lin et al. (2012)
Susceptibility weighted imaging (SWI)	+Can map blood-brain barrier disruption and tau deposition	+Predictive ability limited in adults	Baugh et al. (2012), Kou et al. (2009), Ashwal et al. (2006), and Gavett et al. (2011a)
	+Shown to predict outcome in pediatric patients post-injury	
	+Can detect microhemorrhages with DTI	
Electroencephalography (EEG)	+Components of P300 such as P3a/P3b altered chronically post-concussion	+Minimal investigation outside of boxing	Costanza et al. (2011) and Gavett et al. (2011a)
	+Low cost	+Analysis difficult	

*Each modality exhibits potential strengths and weaknesses, presented here, affecting its viability for further CTE-related research*.

### Diffusion tensor imaging

Diffusion tensor imaging (DTI) represents one of the more thoroughly investigated techniques for detecting axonal injury clinically (Mac Donald et al., 2007; Smits et al., 2011; Shah et al., 2012; Shenton et al., 2012) and has even been shown to correlate with long-term outcome in preclinical rodents models of TBI (Maller et al., 2010). Perhaps most notably, preliminary studies in retired professional athletes subjected to repeat head trauma demonstrate a correlation between history of concussion and DTI measures, particularly in the callosal white matter (Gavett et al., 2011a). How DTI-based findings are altered with age and whether these findings correlate with neurodegenerative and behavioral changes associated with CTE remains to be seen (Tremblay et al., 2012). Regardless, DTI appears to be a promising technique for detecting and tracking structural correlates of repetitive brain injury (Chappell et al., 2008; Bazarian et al., 2012; Bennett et al., 2012; Hutchinson et al., 2012; Li et al., 2012), including those believed to be specific to CTE.

### Functional magnetic resonance imaging

Functional magnetic resonance imaging (fMRI) is unique amongst the various imaging modalities discussed within this work due to the ability to provide insight into functional alterations, particularly working memory, and anatomic or structural changes simultaneously. The capability to measure brain-behavior relationships has proved useful in other neurodegenerative diseases and appears promising for further investigation of CTE-related changes (Gavett et al., 2011a). Based on blood oxygen level dependent (BOLD) signal, fMRI is advantageous in that serial scans can be administered without exposure to harmful ionizing radiation (Sanchez-Carrion et al., 2008). This is particularly useful for fMRI-based studies as baseline measurements are required for comparison due to inter-individual variability (Rosenbaum and Lipton, 2012). Following exposure to head trauma, deficits on fMRI are apparent in both concussive and subconcussive injury groups with concussive injury producing a more severe deficit (Talavage et al., 2010; McDonald et al., 2012). Importantly, studies utilizing both fMRI and ImPACT for neuropsychological testing (NPT) have revealed deficits in working memory, even in the group sustaining only subconcussive impacts, and these deficits appear to be related to the number of impacts rather than the magnitude of any one impact (Breedlove et al., 2012). Furthermore, in a preclinical model of TBI, fMRI response was diminished acutely and correlated with functional deficits based on the forepaw placement test (Henninger et al., 2007). The use of fMRI for assessing the more subtle deficits produced by repetitive subconcussive impacts warrants further investigation, and in particular, longitudinal studies investigating the persistence of deficits on fMRI and the likelihood of further development for detecting, diagnosing, and tracking CTE.

### Magnetic resonance spectroscopy

Magnetic resonance spectroscopy (MRS) is a non-invasive method that measures brain chemistry associated with DAI and neuronal injury (Gavett et al., 2011a; Shenton et al., 2012). It is sensitive for changes occurring with age, mTBI, and mild cognitive impairment (MCI; Tshibanda et al., 2009). As an imaging modality, ^1^H MRS is used to detect changes in *N*-acetylaspartate (NAA), myoinositol (mI), choline, lactate, and adenosine triphosphate (ATP) production in specific ROI. NAA is a neuronal marker and is reported as a NAA:creatinine ratio. Other markers that may prove useful in neural injury include mI, a glial marker (Signoretti et al., 2010), choline-based compounds, indicative of membrane integrity, and lactate, an indirect marker for ischemia (Signoretti et al., 2010). The metabolite levels in an injured brain are compared to levels in a healthy brain for analysis (Vagnozzi et al., 2010). A decrease in NAA and ATP is indicative of reversible neuronal and mitochondrial dysfunction (Henry et al., 2011b). An elevation of the NAA:creatinine ratio indicates persistent damage following mTBI (Signoretti et al., 2011). H_2_O is often measured as an internal control in the patient during imaging (Tremblay et al., 2012). By monitoring the metabolites and H_2_O, the progress of a patient can be tracked longitudinally (Bigler and Maxwell, 2012). It is even possible to detect changes in the brain prior to onset of symptoms such as in subconcussive injuries (Henry et al., 2010). Furthermore, metabolite levels remain altered long after symptoms subside, which gives credence to the idea of long-term degeneration occurring and presence of a latent period (Kleindienst et al., 2005). As MRS utilizes current clinical MR scanners and does not administer ionizing radiation to the patient, MRS may represent an ideal imaging modality for long-term studies requiring repeated measurements. Furthermore, a preliminary study conducted by Lin and colleagues demonstrated persistent changes in Cho and Glx in 5 professional athletes, aged 31–54 years. Specifically, both Cho and Glx were significantly increased as compared to controls (Lin et al., 2010, 2012; Gavett et al., 2011a). Additionally, studies have demonstrated changes in mI consistent with MCI literature that correlate with NFT count in AD brains upon pathological examination (Tremblay et al., 2012). Consequently, understanding the effect of repetitive brain injuries on mI levels over time may be of interest for future investigation due to the strong correlation with episodic memory deficits observed in formerly concussed athletes (Tremblay et al., 2012). Based on this evidence, MRS appears promising for not only elucidation of CTE pathophysiology but also tracking the effects of repetitive brain trauma longitudinally. In this manner, MRS may also be useful for the diagnosis of CTE in effected individuals.

### Positron emission tomography

Positron emission tomography (PET) can be used to detect metabolic rates in a variety of tissues of interest, including the brain (Price and Jones, 1995). While PET has traditionally been used in cases of more severe injury, recent evidence indicates a potential role in mTBI (Mase et al., 2004; Venneti et al., 2007). In boxers, hypometabolism was observed in regions likely affected by impacts to the side of the head such as the portion of the frontal lobe anterior to Broca’s area (Provenzano et al., 2010). Similarly, hypometabolism was observed in the posterior cingulate cortex and posterior parietal lobes (Provenzano et al., 2010). In other patients afflicted with TBI, hypometabolism may be seen commonly in the anterior temoporal lobe and orbitofrontal lobe due to rapid acceleration-deceleration mechanisms (Provenzano et al., 2010). Other promising developments in PET imaging include the development of ligands specific for disease states such as AD. For example, Pittsburgh compound B selectively binds Aβ whereas others under development non-selectively bind both Aβ and tau (Gavett et al., 2011a). While Aβ is not diagnostic for CTE, this technology appears potentially promising for the imaging of CTE should tau-specific compounds be identified. Furthermore, recent studies using F^18^ DDNP glucose-PET, conducted by Dr. Gary Small at UCLA, show promise for identifying tau protein deposition in the form of NFT’s in subjects with potential CTE (Wagner, 2012). F^18^ DDNP glucose-PET allows for labeling of both plaques and tangles, as has been shown in cases of MCI as well as AD, making this technique exceptionally promising in identifying characteristic pathological features of CTE as they develop pre-mortem (Small et al., 2006). This effort is significant in that it could potentially allow, for the first-time ever, the diagnosis of CTE pre-mortem (Small, 2012). Similarly, identification of NFT’s following brain injury would provide substantial insight into the timing of development and pathophysiology of CTE.

### Single photon emission computer tomography

Single photon emission computer tomography (SPECT), similar to PET in the use of radionuclides, can be used for measurement of cerebral perfusion and has been shown to detect abnormalities in 41% of amateur boxers in comparison to 14% of controls. Furthermore, abnormalities were generally singular in the control group yet multiple in the group of boxers subjected to repetitive brain trauma (Kemp et al., 1995). While SPECT has not been utilized in studies of CTE, CTE is associated with amyloid protein deposition in a significant number of cases and amyloid is notably largely perivascular in deposition, particularly in regions of abnormal vasculature, which could account for abnormal findings on SPECT (Kemp et al., 1995). It has also been correlated with functional deficits revealed on NPT. Specifically, NPT strongly predicts diminished perfusion on SPECT but the inverse is not true (Lin et al., 2012). The reasons for this mismatch are currently unknown but may be due to SPECT being obtained at a resting state while NPT requires neural activity and processing (Lin et al., 2012). In spite of this lack of correlation and lack of specificity when considering other disease conditions, SPECT has clear value in recovery prognostication based on previously reported findings (Lin et al., 2012).

### Susceptibility weighted imaging

Susceptibility weighted imaging (SWI) is used to detect microhemorrhages, the intact structure of the venous system, and oxygen saturation following neurotrauma (Shen et al., 2007). Microhemorrhages, venous thrombosis, and ischemia are common secondary injuries following mTBI (Aiken and Gean, 2010). Furthermore, the extent of blood-brain barrier (BBB) disruption and perivascular tau deposition can be mapped with SWI (Baugh et al., 2012). Tau accumulates in the brain during the progression of CTE (Stern et al., 2011). The imaging technique works by taking phase data from a *T*2* weighted MRI and creating a second mapped image. This image allows both blood oxygen levels and the amount of deoxyhemoglobin to be measured (McCrea et al., 2008). It also localizes concentrations of paramagnetic iron and detects areas of iron translocation (Cheng et al., 2010). Iron deposition may play a role in CTE symptom manifestation (Zhang et al., 2010). The images produced with SWI are highly functional, velocity-compensated, and gradient echoed (Meoded et al., 2012). When used with DTI, SWI can accurately show which areas of white matter are damaged by microhemorrhages (Kou et al., 2009). This modality offers a unique tool to detect areas of injury early, and provides clinicians with information on how to manage the care of the patient, particularly in children in which SWI has been shown to predict future outcomes following injury (Ashwal et al., 2006; Gavett et al., 2011a). Unfortunately, the predictive ability is more limited in adults at present but necessitates further study (Gavett et al., 2011a).

### Electroencephalography

Electroencephalography (EEG) has been used to demonstrate mental processing impairment in 12 professional concussed boxers based on increased P300 latency and lessened amplitude. P300 has been identified as a cognitive event-related potential (ERP) with a novel amplitude and latency, making it a potentially suitable measure of brain processing ability. Similarly, investigators have demonstrated altered components of P300, such as P3a/P3b, decades after concussion in older athletes with a history of past concussion compared to controls (Costanza et al., 2011; Gavett et al., 2011a). Based on these findings, EEG may have utility in assessing long-term cognitive effects associated with concussion and therefore, may be useful in identifying those predisposed to CTE development.

### Future directions

Advances in imaging technology and improved access provide nearly limitless opportunities for both understanding the pathophysiology and diagnosis of CTE pre-mortem. As such, it may be possible to initiate both treatments and preventive measures prior to emergence of CTE. Many challenges remain such as clarifying the role of subconcussive injury in CTE development and how to most readily identify suspected subconcussive injuries on the athletic or battlefield. By utilizing techniques such as MRS and fMRI, pathology can be correlated with functional deficits and hopefully lead to more appropriate identification and monitoring of those with suspected injuries, regardless of severity. Other challenges such as the need for baseline measurements remain to be overcome as well but clearly warrant additional investigation.

## Biomarkers for CTE: A New Frontier

As discussed in previous sections modern imaging techniques are one way by which diagnosing and tracking the progression of CTE may be feasible in the near future. Unfortunately, access and economic issues associated with imaging techniques may somewhat limit their ultimate usefulness in the diagnosis of CTE, especially during the acute phases of injury. With CTE potentially being a large public-health issue, a simple and cost effective manner to diagnose and possibly track progression of the disease is crucial (Baugh et al., 2012).

Biomarkers represent one method by which CTE may one day be diagnosed. Additionally, it may be possible to track disease severity and progression through the use of a biomarker(s). Ideally, a potential biomarker for CTE would be minimally invasive, have diagnostic potential and would correlate to disease severity, allowing care providers to track progression of the disease. It should be sensitive enough to detect the disease and when used in conjunction with clinical evidence of CTE symptoms and a past history of repeated head injuries, allow for a diagnosis. As described above, there is currently no accepted method of diagnosing CTE until post-mortem pathological analysis has been conducted (Baugh et al., 2012). A readily available biomarker with the aforementioned traits would give practitioners a useful tool for the diagnosis and tracking of patients suffering from CTE.

Currently there is a paucity of studies aimed at determining a biomarker(s) specifically for CTE. Many studies aimed at elucidating biomarkers for TBI and other types of neurotrauma have been conducted. One difficult aspect of searching for a CTE biomarker is that CTE symptomology can be similar to a variety of other neurologic disorders. This may add a layer of complexity to conducting human clinical studies in an attempt to discover suitable biomarkers. The neuropathology of CTE, however, is not distinctly different, as hyperphosphorylated tau and TDP-43 deposition are found in a variety of neurodegenerative diseases outside CTE (McKee et al., 2010; Baugh et al., 2012; Saulle and Greenwald, 2012). Additionally, recent reports indicate that CTE may be associated with symptomology similar to ALS, further complicating the likelihood of clinical diagnosis without improved diagnostic tests (McKee et al., 2010). These reports emphasize the need for a biomarker with high specificity that can delineate CTE from other neurologic disorders.

There are two primary body fluids where readily attainable biomarkers of CTE may be located. These include the cerebral spinal fluid (CSF) and the blood. Blood or plasma samples have the distinct advantage of being minimally invasive and easy to obtain. CSF samples, however, are in more direct contact with the CNS and may confer an advantage in this regard to the determination of suitable biomarkers for CTE. Routine use of CSF in the diagnosis or tracking of CTE presents several obvious problems associated with obtaining CSF samples. Therefore, ideally a biomarker aimed at detecting CTE in the periphery would be found in serum or plasma samples obtained via venipuncture. As discussed above, currently there is a lack of primary studies aimed at the elucidation of biomarkers in either one of these body fluids for CTE. However, several studies have been conducted in the realm of TBI and mTBI and these will be discussed herein.

It may be possible that biomarkers in development for TBI may be useful during the acute injury phase of patients at risk for CTE. The repeated concussive and subconcussive blows that result in CTE may alter levels of particular biomarkers studied for TBI immediately following these events. The tracking of these acute changes, although not specific to CTE, may give healthcare professionals insight into those patients at the greatest risk for developing CTE later in life. Equally important though, is the potential utility for biomarker surveillance in patients with known or suspected post-concussion syndrome.

Many studies that have been conducted aimed at determining a biomarker for TBI or mTBI in the plasma or serum. A study conducted by Mondello et al. has determined that glial neuronal ratio (GNR), characterized as the ratio between glial fibrillary acidic protein (GFAP) and ubiquitin carboxy-terminal hydrolase-L1 (UCH-L1) in the serum of TBI patients is characteristic of the type of TBI injury, focal vs. diffuse (Mondello et al., 2012). It was noted by Mondello et al. (2012) that their studies may have implications for diagnosis in TBI patients in the early acute phases of injury, however whether these results would have implications for CTE patients has yet to be determined. It is possible that after multiple concussive or subconcussive blows to the head that the GNR may be altered and may offer insight into the extent of injury and potential for later development of CTE.

Several other studies aimed at elucidating valuable biomarkers for TBI in the serum have been conducted and may offer insight into the production of a biomarker(s) specific to CTE. It has been reported that plasma bilirubin levels in TBI patients are elevated on days subsequent to injury (Dohi et al., 2005). Unfortunately, this study only determined bilirubin levels up to 4 days post-injury. The determination as to whether the increases in plasma bilirubin remain elevated for a significant amount of time post-injury or whether it is specific to the acute injury phase has yet to be made. Additionally, the determination as to whether bilirubin concentrations in the plasma would be elevated in patients at risk for CTE has also yet to be made.

Another intriguing potential serum biomarker for CTE may be the protein neuron-specific enolase. Zetterberg et al. (2009) have shown that this protein is increased in the serum of boxers as compared to healthy controls after the boxers abstained from boxing for a period of 2 months. Interestingly, elevated levels of neuron-specific enolase were detected 2 months after a non-participatory period of boxing, however other biomarker candidates for CTE such as S-100B, brain-derived neurotrophic factor (BDNF), and heart-type fatty acid binding protein (H-FABP) were found to be unchanged (Zetterberg et al., 2009). These results indicate that neuron-specific enolase may be one protein biomarker that has the potential for use as a diagnostic tool. It is possible that neuron-specific enolase remains elevated for an extended time period after injury and may be a useful tool for diagnostic purposes in athletes and patients who have suffered multiple concussive and subconcussive blows to the head.

An area of biomarker research that has received much attention is the field of microRNA. It is possible that potential biomarkers for CTE may not only be protein-based in nature. A recent report by Balakathiresan et al. (2012) has shown that levels of microRNA let-7i are elevated post-blast-induced TBI in both the CSF and the serum of rats exposed to a model of this type of TBI. Additionally, this group showed that the expression of five microRNA’s were altered post-blast TBI in the serum of these animals (Balakathiresan et al., 2012). These included let-7i, miR-122, miR-200b, miR-340-5p, and miR-874 (Balakathiresan et al., 2012). Further study is needed in both animal models, as well as humans, to validate the potential for using microRNA’s as potential biomarkers for TBI and possibly CTE. Although this study was conducted using a blast TBI animal model, a recent report has shown that this model produces pathology similar to that seen in CTE and models similar to this may be viable animal models for the study of CTE (Goldstein et al., 2012). The production of validated animal models could greatly aid in the discovery of biomarkers aimed at detecting CTE and the production of potential clinical therapies for treating CTE. Other studies have also successfully identified RNA-based biomarkers for mTBI based on both micro- and snoRNA in peripheral blood mononuclear cells (PBMCs). Using a panel of three markers, veterans that experienced mTBI were detected with 89% accuracy, 82% selectivity, and 78% specificity (Pasinetti et al., 2012). Future studies utilizing both animal models as well as clinical samples may reveal additional RNA-based molecules that may be viable biomarkers for detecting and possibly tracking CTE.

One particularly interesting report recently published, studied a variety of biomarkers aimed at detecting TBI and mTBI in Olympic boxers with an extended history of many bouts (Neselius et al., 2012). This study determined extent of increases in neurofilament light protein (NFL), total tau (T-tau), tau phosphorylated at threonine 181 (P-tau_181_), H-FABP, GFAP, S-100B, and the 42 amino acid form of amyloid β (Aβ1–42) in Olympic boxers both in the acute phase after a bout and an extended phase of 2 weeks post-bout and compared them to non-boxing controls. It was found that both NFL and GFAP levels within the CSF were significantly different from controls in both the acute and extended phase of testing (Neselius et al., 2012). The determination as to whether either of these correlated to injury severity was unable to be conclusively made in this study (Neselius et al., 2012).

However, a previous study has shown that there may be a potential correlation between NFL levels in the CSF and injury severity (Zetterberg et al., 2006). Zetterberg et al. (2006) showed that NFL, T-tau, and GFAP were increased in the CSF of boxers and the levels appear to be correlated to injury severity, as boxers who received more hits in number or severity had increased levels of these markers. Only NFL in these studies however, was increased for an extended time period (tested at 3 months) as compared to controls (Zetterberg et al., 2006). The extended increase in NFL levels within the CSF post-injury may make it a viable candidate for a biomarker for mTBI and CTE however whether levels of NFL within the CSF correlate to later disease severity in the case of CTE or mTBI has yet to be conclusively made. NFL levels however, have been shown to be altered in other types of neurologic trauma and disorders so this may ultimately decrease its viability as a tool for detecting, diagnosing, and tracking CTE disease progression (Avsar et al., 2012; Tortelli et al., 2012).

Although levels of proteins such as NFL and tau may not be specific with regards to CTE, a battery of various biomarker proteins combined with patient history and imaging studies (discussed in previous sections) may result in the ability to make a pre-mortem diagnosis of CTE via a reference fractional laboratory index using a high throughput analytical system. In this respect, a combined battery of proteins such as tau, GFAP, NFL, S100-B, and bilirubin may offer more accurate quantitative data with regards to a patients risk for developing CTE and when combined with other modalities of relevant clinical data, may be used as even more accurate diagnostic indicators at some point in the future.

## Linking Pathophysiology to Imaging and Biomarker Discovery

Perhaps the greatest challenge concerning development of biomarkers or imaging modalities for prevention, diagnosis, and prognosis of CTE is the current lack of well-elucidated disease pathophysiology. Consequently, few clearly identifiable and specific disease markers have been identified. Without first understanding disease development more clearly, likely via both clinical research and utilization of animal models of repetitive brain trauma, logical discovery of CTE-associated markers appears unlikely. Recent work has demonstrated the presence of CTE-like neuropathology in wild-type mice following single-blast exposure and increased formation of NFTs in a transgenic mouse model following repetitive injury (Yoshiyama et al., 2005; Goldstein et al., 2012). In a single-injury experiment, again using transgenic mice utilized in AD models, tau and amyloid-β accumulation was accelerated (Tran et al., 2011). Similarly, studies have shown the presence of phosphorylated tau and amyloid-β months after initial fluid percussion injury in the genetically unmodified rat (Hoshino et al., 1998).

Despite the present lack of clear elucidation of disease pathophysiology, certain imaging techniques such as MRS, fMRI, and tau-specific labeling compounds in PET, have begun and will likely continue to provide insight into CTE development via longitudinal changes in neurologic function and health via a variety of markers. Similarly, work in other disease conditions that allows for imaging of activated microglia, consistent with the notion of immunoexcitotoxicity, may provide further insight into disease development and progression (Venneti et al., 2007).

In addition to assisting in the diagnosis and prognosis of patients suffering from CTE, continued improvements in imaging and biochemical assays associated with CTE identification may promote risk factor identification and provide insight into periods of increased risk. For example, the role of age at time of impact and associated brain trauma in disease development is unclear. Understanding this issue warrants further investigation as neuropathologic changes associated with CTE have been found in an asymptomatic 18-year-old high school football player and consequently, age at time of first head injury may contribute to CTE incidence (Saulle and Greenwald, 2012).

## Conclusion

**Figure 1:**
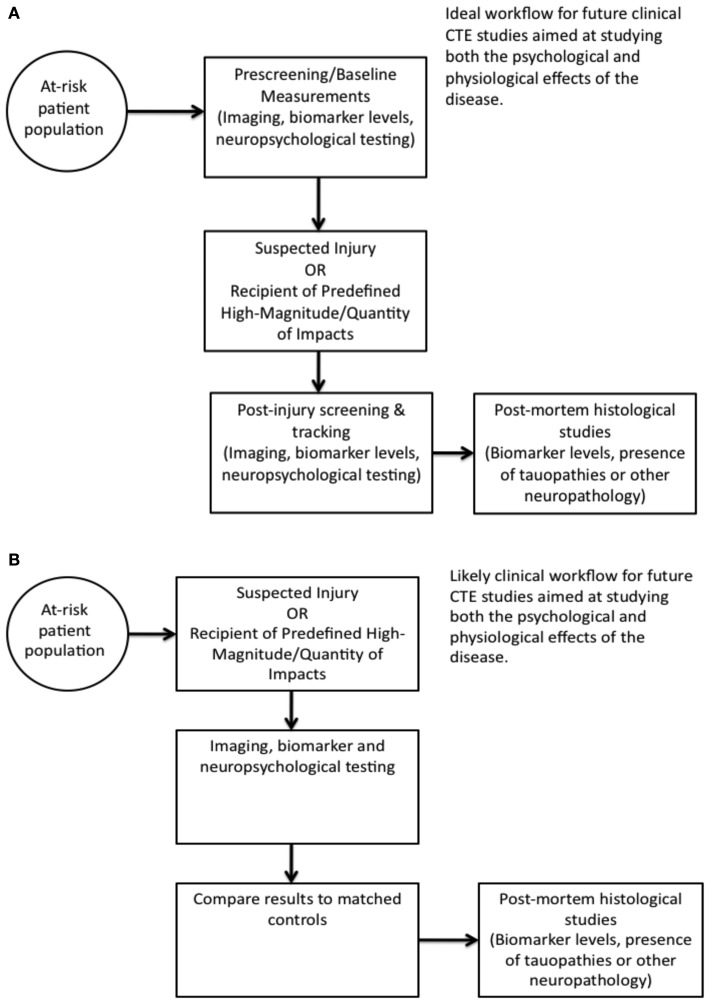
**Imaging and biomarker studies may be useful in improving understanding of the role of repetitive concussive and subconcussive injury in disease development**. Two experimental paradigms are presented representing a potentially more ideal, but costly, scenario **(A)** and the more cost-conscious approach **(B)**.

Chronic traumatic encephalopathy is an emerging public-health concern due to disease prevalence likely higher than previously recognized and continued popularity of contact sports as well as involvement in military combat that places soldiers at risk for explosive blasts and subsequent TBI. As CTE represents a devastating deterioration of neurologic function, a clear need for improved diagnostic and prognostic tests exists. To accommodate this need, as well as more clearly elucidate disease pathophysiology, imaging, and/or biomarker-based tests are required. We propose a potential work-flow for implementation of both imaging and biomarker-based studies for improving the understanding of the role concussive and subconcussive impacts play in long-term disease development (Figure 1). While the specifics remain open to interpretation, obtaining pre-injury exposure studies as well as after a suspected or confirmed injury parameters is likely to provide further insight into the effects of TBI. It is only by more accurately identifying CTE pre-mortem and studying disease progression and mechanisms that we can establish improved therapeutic approaches.

## Conflict of Interest Statement

The authors declare that the research was conducted in the absence of any commercial or financial relationships that could be construed as a potential conflict of interest.
